# Compartmental models for seasonal hyperendemic bacterial meningitis in the African meningitis belt

**DOI:** 10.1017/S0950268818002625

**Published:** 2018-09-28

**Authors:** T. Koutangni, P. Crépey, M. Woringer, S. Porgho, B. W. Bicaba, H. Tall, J. E. Mueller

**Affiliations:** 1 Université Pierre et Marie Curie, 4 Place Jussieu, 75005 Paris, France; 2 Unité de l'Epidémiologie des Maladies Emergentes, Institut Pasteur, 25-28 Rue du Dr Roux, 75015 Paris, France; 3 EHESP French School of Public Health, Sorbonne Paris Cité, 20 avenue George Sand, 93210 La Plaine St Denis, France; 4 UMR Emergence des Pathologies Virales, Université Aix-Marseille – IRD 190 – Inserm 1207 – EHESP, 27 Boulevard Jean-Moulin 13385 Marseille Cedex 5, France; 5 Univ Rennes, EHESP, REPERES (Recherche en pharmaco-épidémiologie et recours aux soins) – EA 7449, F-35000 Rennes, France; 6 Institut de Biologie de l'Ecole Normale Supérieure (IBENS), PSL Research University, 45 Rue dʼUlm, 75005 Paris, France; 7 Direction de la Lutte contre la Maladie, Ministère de la Santé, 03 BP 7035 Ouagadougou 03, Burkina Faso; 8 Agence de Médecine Préventive, 10 BP 638. Ouagadougou, Burkina Faso

**Keywords:** Meningitis, mathematical model, African meningitis belt, hyperendemicity, seasonality

## Abstract

The pathophysiological mechanisms underlying the seasonal dynamic and epidemic occurrence of bacterial meningitis in the African meningitis belt remain unknown. Regular seasonality (seasonal hyperendemicity) is observed for both meningococcal and pneumococcal meningitis and understanding this is critical for better prevention and modelling. The two principal hypotheses for hyperendemicity during the dry season imply (1) an increased risk of invasive disease given asymptomatic carriage of meningococci and pneumococci; or (2) an increased transmission of these bacteria from carriers and ill individuals. In this study, we formulated three compartmental deterministic models of seasonal hyperendemicity, featuring one (model1-‘inv’ or model2-‘transm’), or a combination (model3-‘inv-transm’) of the two hypotheses. We parameterised the models based on current knowledge on meningococcal and pneumococcal biology and pathophysiology. We compared the three models' performance in reproducing weekly incidences of suspected cases of acute bacterial meningitis reported by health centres in Burkina Faso during 2004–2010, through the meningitis surveillance system. The three models performed well (coefficient of determination *R*^2^, 0.72, 0.86 and 0.87, respectively). Model2-‘transm’ and model3-‘inv-transm’ better captured the amplitude of the seasonal incidence. However, model2-‘transm’ required a higher constant invasion rate for a similar average baseline transmission rate. The results suggest that a combination of seasonal changes of the risk of invasive disease and carriage transmission is involved in the hyperendemic seasonality of bacterial meningitis in the African meningitis belt. Consequently, both interventions reducing the risk of nasopharyngeal invasion and the bacteria transmission, especially during the dry season are believed to be needed to limit the recurrent seasonality of bacterial meningitis in the meningitis belt.

## Introduction

Africa has the highest contribution to the global burden of bacterial meningitis, a severe disease with up to 30% case fatality despite timely antibiotic treatment and 20% of survivors living with psychomotor sequelae [1–4]. In the African meningitis belt spanning the Sahel from Senegal to Ethiopia [5], meningococcal and pneumococcal meningitis incidence displays a seasonal pattern during the dry season (December through May) with a 10- to 100-fold increase of weekly incidences at local health centre, district and national levels, which subsides with the onset of the rainy season [6, 7]. This seasonal increase in the disease incidence in the dry season is observed every year and consistent across countries of the so-called African meningitis belt: a situation commonly described as ‘ubiquitous seasonal hyperendemicity’. In addition, localised epidemics of meningococcal meningitis occur unpredictably limited to one or few villages, with attack proportions beyond 1% [1]. Despite introduction of effective and affordable conjugate vaccines against meningococcal serogroup A (in December 2010) [8] and 10–13 pneumococcal serotypes (in 2013) [9] through mass vaccination campaigns and infant routine immunisation, respectively, this pattern continues, mainly due to the persistence of other epidemic meningococcal serogroups and high adult pneumococcal meningitis incidence.

A distinction between the mechanisms underlying meningitis ubiquitous annual seasonality (hyperendemicity) and localised epidemics would have implication on how the disease is mathematically modelled and how control strategies are designed in the meningitis belt [1, 6, 7]. A better understanding of the mechanisms behind this epidemiology is therefore needed, along with appropriate mathematical models allowing the identification of optimised preventative vaccination strategies.

Previous modelling efforts relied on a wide range of unknown parameters values [10] given the lack of surveillance data from which parameters could be estimated. Others have used incidence data for model fitting at low spatial resolution, mainly data aggregated at district level [11, 12]. This does not allow differentiating between dry seasons with localised epidemics and dry seasons without localised epidemics, as localised epidemic usually can be seen at the health centre level only [13, 14]. To go further from these previous efforts, we have developed a model in which unknown parameters values are estimated based on meningitis surveillance data at a fine spatial (health centre) and temporal (weekly) scale. This study focuses on modelling the regular seasonal hyperendemicity, observed during all dry seasons across the meningitis belt and used surveillance data from Burkina Faso for parameters estimation and model validation. Burkina Faso lies within the meningitis belt with an enhanced surveillance system for bacterial meningitis.

Two main explanations have been suggested for the hyperendemic incidence increase during the dry season. First, the climatic conditions such as low relative air humidity and high aerosol load experienced across countries of the meningitis belt during the dry season (November through May) could damage the nasopharyngeal mucosa and thus facilitate invasion of meningococci and pneumococci into nasopharyngeal tissues, which results in meningitis [15]. The second hypothesis suggests that these climatic conditions or related behavioural changes could facilitate the bacterial transmission in the population and thus proportionally increase disease incidence [15]. Mueller and Gessner's hypothetical explanatory model builds on the first hypothesis (increased invasion rate) [16].

In a systematic review and meta-analysis of published data from the meningitis belt [7], seasonal hyperendemicity of meningococcal meningitis was associated with a seasonal increase of the case–carrier ratio, while the prevalence of meningococcal carriage assessed in cross-sectional carriage studies did not change with season, thus supporting the first hypothesis. However, in a multi-site series of cross-sectional meningococcal carriage studies, Kristiansen *et al*. [17] reported minor but statistically significant changes in serogroup A *meningococcal* carriage prevalence between the rainy and dry season (from 0.24% to 0.62%), a finding supporting the second hypothesis (increased transmission rate). The present study aimed at using mathematical models to assess which of these competing hypotheses or their combination best explained observed hyperendemic incidence pattern of suspected bacterial meningitis in Burkina Faso.

## Methods

### Study setting and surveillance data

In countries of the meningitis belt, suspected cases of bacterial meningitis (as defined by the WHO) are systematically notified from the peripheral level (local health centres) to the intermediate (district) and central (national) levels since the establishment of an enhanced meningitis surveillance network in 2003 across the meningitis belt with the support of the WHO. Suspected meningitis cases are notified from the local health centres on a weekly basis and the number of cases must be reported even when there is zero case at all levels. Burkina Faso is one of the countries entirely located within the meningitis belt for which we had access to weekly counts of suspected bacterial meningitis cases at the health centres level. In the country, prior to 2010, suspected meningitis case notification was often supplemented by laboratory investigation of a subset of the notified cases; especially when epidemic threshold defined at the district level is crossed, to guide epidemic preparedness and choice of polysaccharide vaccine. Acute bacterial meningitis in the meningitis belt is most commonly caused by *Neisseria meningitidis*, *Streptococcus pneumoniae* and, since introduction of a conjugate vaccine, to a lower extent *Haemophilus influenzae* Type b [18, 19]. Suspected and laboratory-confirmed cases correlate well usually [20] and suggest a relatively good performance of the surveillance system and appropriateness of the data for epidemiologic studies. Until 2010, and before the introduction of serogroup A meningococcal conjugate vaccine in December 2010, meningitis epidemics were predominantly caused by *N. meningitidis* across the belt. Pneumococcal meningitis contributes to meningitis hyperendemicity and mimics the seasonality of meningococcal meningitis across the meningitis belt [21]. In this study, to estimate the unknown parameter values and to evaluate our models performances, we used data from routine surveillance of suspected acute bacterial meningitis cases recorded from 2004 through 2010 in health centres in Burkina Faso (a period preceding introduction of the MenAfrivac serogroup A meningococcal vaccine). While data aggregated at the district level are available in routine surveillance reports, this database of original weekly health centre data had been compiled in a collaborative effort between the Direction de la Lutte contre la Maladie (DLM) of the Ministry of Health of Burkina Faso, EHESP French School of Public Health, and the Agence de Médecine Préventive (AMP), Paris, France. We selected four health districts (Houndé, Lena, Karangasso Vigué and Séguénéga) for the completeness of data, providing 126 health centre years. Seasonal hyperendemicity and localised epidemics are two distinct phenomena involving potentially different mechanisms [16]. Therefore, we separated health centre years with localised epidemics from those with usual hyperendemic incidences, using the threshold definition of 75 weekly cases per 100 000 maintained during at least two consecutive weeks [13]. Thus, only hyperendemic health centre year curves are used for models’ analysis in this study. Seasonal hyperendemicity of bacterial meningitis is a regular phenomenon observed every year in the belt. Localised meningitis epidemics are irregular in the meningitis belt. Therefore, we considered a deterministic framework as a reasonable first step over a stochastic framework in modelling hyperendemic meningitis in the belt. Overall, 64 hyperendemic health centre years (out of the 126) identified based on the defined threshold were used in the primary analysis (Supplementary Fig. S1–S3).

A second threshold of 50 weekly cases per 100 000 maintained during at least two consecutive weeks was used for sensitivity analyses. This sensitivity analysis was performed to assess the efficiency of the model when using a lower incidence threshold definition of hyperendemic incidence excluding health centre years with outlier peak incidence from the primary analysis. Fifty-seven out of the initial 64 hyperendemic health centre years were then identified and used in the sensitivity analysis. We smoothed incidence time series using a simple moving average on a 3-week window to reduce random noise in the data and the influence of instable estimates of incidence potentially due to delays in reporting. We used the SMA function in the TTR R package to achieve this.

### Model structure

**Fig. 1.**
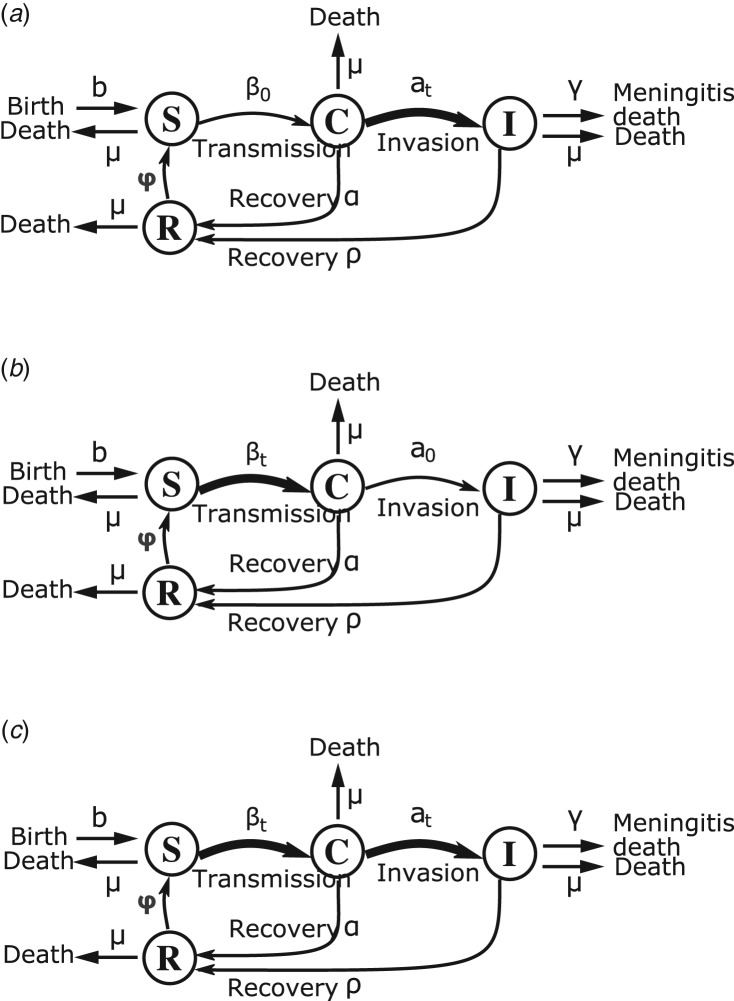
Flow chart of state progression of individuals between the different epidemiological classes of the SCIRS models. Thick black arrows indicate parameters with seasonal forcing. (a) Model1-‘inv’: seasonal forcing of the invasion rate alone, (b) model2-‘transm’: seasonal forcing of the transmission rate alone, (c) model3-‘inv-transm’: seasonal forcing of the transmission and invasion rate.

Similar to Irving *et al*. [10], we used a compartmental deterministic Susceptible–Carrier–Ill–Recovered–Susceptible (SIRS) model, which divides the population into four mutually exclusive groups (Fig. 1): individuals susceptible to infection (*S*); asymptomatic carriers (*C*) who can transmit the bacteria (meningococci or pneumococci) to susceptibles; individuals ill from meningitis (*I*) following contagion and who are also infectious; and individuals who have recovered (*R*) from asymptomatic carriage or meningitis. Recovered individuals have developed temporary immunity and become susceptible once immunity has waned [22]. Transition rates include rates for birth, natural death and death from meningitis (Table 1). The system of ordinary differential equations defining the model dynamic is as follows:1
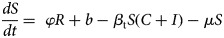
2
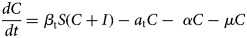
3
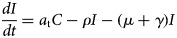
4
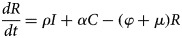
5
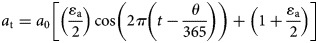
6
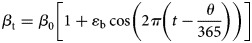

**Table 1.** Fixed and unknown parameters values and ranges for calibration of the models of seasonal hyperendemic bacterial meningitis in the African meningitis belt

Parameter	Short description	Plausible range	Initial value^a^	Unit	Comments and sources
Unknown parameters					
* β*_0_	Meningococcal mean transmission rate	>0	0.5	Day^−1^	Unknown. Only positive values
* a*_0_	Meningococcal mean invasion rate given carriage	0.002–0.012	0.007	Month^−1^	Inferred from case–carrier ratios estimated in a systematic review, specific for season and epidemiological context [7]
*α*	Rate of loss of carriage	1–52	12	Year^−1^	Unknown, carriage duration between 1 week and 1 year, range inferred from [20, 23]
*φ*	Rate of loss of natural immunity	0.2–12	4	Year^−1^	Unknown, persistence of natural immunity of between 1 month and 5 years, range inferred from [20, 24]
*ε*_a_	Amplitude of seasonal forcing of invasion rate	0–100	50		An amplitude of 0 means that the baseline invasion rate remains constant across seasons; of 100 means it increases up to 100-fold
*ε*_b_	Amplitude of seasonal forcing of meningococcal transmission rate	0–1	0.5		An amplitude of 0 means that the baseline transmission rate remains constant across seasons, and values up to 1 means presence of seasonality
*θ*	Calendar day of maximal invasion rate	91–112	97		Assuming correlation with aerosol load during period of relative humidity <40% (calendar week 13 through 16) [25]
*S*_0_	Proportion of initial susceptibles in the population	0–1	0.5		The proportion of susceptible at the beginning of the calendar year (1 January)
*C*_0_	Proportion of initial carriers in the population	0–1	0.01		The proportion of carriers at the beginning of the calendar year (1 January)
Fixed parameters values					
*γ*	Death rate from meningitis	5.2		Year^−1^	Case fatality = 10% [1]
*μ*	Natural death rate	0.02		Year^−1^	Life expectancy = 54 years [26]
*ρ*	Recovery rate	52		Year^−1^	Acute phase of bacterial meningitis disease lasts a week on average [27]
*b*	Birth rate	*b* = *μ* + *γI*		Year^−1^	Scaled to keep total population size constant

a Values used as initial values for parameters optimisation routine.

Variables *S*, *C*, *R*, and *I* are proportions of the total population at time *t* in the respective compartments of the model. The models’ parameters are described in Table 1.

### Seasonality

To represent the two hypotheses of increased invasion or transmission rate during the dry season, we included seasonal forcing of the transition rate to invasive disease given carriage (model1-‘inv’), or the bacterial transmission rate (model2-‘transm’), or both (model3-‘inv-transm’). The invasion and transmission parameters (*a*_t_ and *β*_t_) were represented with periodic sinusoidal functions (equations 5 and 6). Based on the explanatory model by Mueller and Gessner [16], and the systematic review of season-specific case–carrier ratio in the meningitis belt [7, 16], the case–carrier ratio (a proxy for the risk of invasive meningitis given colonisation) could increase up to 100-fold during the dry season. We included this information by parameterizing the periodic function of the invasion rate such that variations of up to 100-fold are possible in the dry season depending on the seasonal forcing amplitude (*ε*_a_) estimate which can take on values from 0 to 100. The seasonal forcing amplitudes *ε*_a_ and *ε*_b_ dictate the magnitude of seasonal variation of the invasion and transmission rate, respectively (equations 5 and 6).

### Model assumptions

The model structure assumed a steady and well-mixed population with frequency-dependent transmission. Age structure of the population was deliberately not included in this proof of concept. However, the potential effects of heterogeneous mixing were explored in complementary analyses. Immunity from asymptomatic carriage and disease was assumed temporary. We assumed immunity provided by carriage and disease to be of similar duration, and asymptomatic carriers are as likely as ill individuals to transmit the infection to a susceptible. Ill individuals may be at a greater risk to transmit only from vomiting but are usually bound to bed.

### Parameterisation

We obtained parameters values including natural death rate, death rate from meningitis, recovery rate after bacterial meningitis and birth rate from the scientific literature (Table 1). Case fatality rates of 10–15% were reported during serogroup A epidemics meningitis in the meningitis belt [1]. We inferred natural death rate as the inverse of life expectancy at birth (average life expectancy was 54 years in Burkina Faso) [26], and the average recovery rate as the inverse of duration of acute phase of meningitis (acute phase of bacterial meningitis would last a week on average) [27] (Table 1). Parameters that are not available in the literature were estimated using suspected bacterial meningitis cases report data from Burkina Faso; a country within the meningitis belt. The data consist of weekly counts of new suspected cases of bacterial meningitis recorded at health centres of four districts of the country from 2004 to 2010 together with the population sizes covered by each health centre. The estimated parameters were: the average meningococcal transmission and invasion rates, the amplitudes of seasonal forcing of transmission and invasion rates, the rate at which asymptomatic carriers and ill individuals recover, the duration of temporary immunity and the timing of weekly incidence peak relative to January 1. Initial susceptibles and carriers population size at the start of calendar years were also estimated for each health centre year hyperendemic's curves, as they could not be inferred directly from the literature. We limited the space of potential parameters values to be tested to plausible values according to the published literature if possible (Table 1). For example, we used the 95% confidence interval of the meningococcal case–carrier ratio estimate during the dry hyperendemic season in the meningitis belt [7] as plausible values range for the average bacterial invasion rate (*a*_0_). We estimated all unknown parameters values using a maximum likelihood approach. For each model, parameters values were selected to maximise the Poisson likelihood of observed bacterial meningitis incident cases. We used the COBYLA algorithm, a derivative-free optimisation algorithm, implemented in the R package nloptr for parameters optimisation routine [28]. We chose this algorithm as it is relatively fast, it allows good convergence of the coefficients estimated on our data and it supports optimisation constrains such as parameter range. Several initial values were tested, and best-fit parameters estimates were obtained after 40 000 iterations. Implementations details of the optimisation routine are provided in Supplementary Material S1. In the complementary exploratory analysis investigating heterogeneous mixing of the population age groups in the models, we inferred the effective contact matrix from age-specific force of infection estimates in dry season with ‘minor epidemics’ as reported by Tartof *et al*. [11] in Burkina Faso.

### Model simulation and evaluation

We implemented and simulated the models using R statistical computing software [29], and the lsoda function (deSolve package) for numerical integration of the ordinary differential equations with 1-day time step. We computed weekly incidence as:7
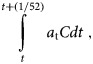
with *a*_t_*C*, the proportion of asymptomatic carriers who becomes ill at time *t*.

We quantitatively assessed the models’ performance accuracy using the coefficient of determination (*R*^2^), the per cent bias (PB), and the ratio of the root-mean-squared-error (RMSE) to observation standard deviation (RSR) (Supplementary Material S1). These three statistics quantify errors in models’ predictions. PB computes the average absolute bias in model predictions of observations. It gives an indication on whether the model results are consistently under- or overestimated compared with the observations [30]. The optimal value of PB is 0.

RSR standardises the RMSE using the observations standard deviation. It incorporates the benefits of error index statistics and includes a scaling/normalisation factor, so that the resulting statistic can be compared across data with different variance. The lower RSR, the better the model simulation performance. We also compared carriage prevalence predicted by the models with carriage prevalence reported by series of meningococcal carriage studies and a review of carriage during wet endemic and dry hyperendemic seasons in the meningitis belt [7, 17, 31]. We assessed the models’ performance qualitatively by visual inspection of trajectories matching plots of model predictions of weekly incidence and observed data, and the ability of the models to fit data across all health centre years with a relatively good accuracy, i.e. capture both the seasonal trend in data, as well as timing and amplitude of observed seasonal peaks. Finally, the three models were compared based on their Akaike Information Criteria (AIC) to account for model complexity associated with the number of input parameters. The lower the model's AIC, the better and an absolute difference in AICs between 0 and 2 was considered weak to distinguish two models.

### Uncertainty and parameter sensitivity analysis

The Latin Hypercube Sampling (LHS) uncertainty technique [32] was used to assess the model robustness to varying fixed and estimated parameters values (uncertainty analysis). Primarily, we evaluated the effect of parameters estimates uncertainty on predictions of the annual cumulative meningitis incidence and the annual average asymptomatic carriage prevalence. The estimates of these two models’ state variables were obtained from the results of uncertainty analyses, and their distribution described for each model. Probability distribution functions (pdfs) of the estimated parameters were unknown. Therefore, we set the parameters pdfs to the uniform distribution. We also set the minimum and maximum values of the uniform distributions to be the 1st and 3rd quartiles of each of the estimated parameters distribution per model. Models were simulated with each of 1000 sets of parameters values sampled based on the LHS schema. We sampled a large number of values (1000) without replacement, within the boundaries of each parameter space to ensure that a great number of plausible parameters values combinations were explored. We calculated partial rank correlations coefficients (PRCC) between each of the estimated parameters and the sensitivity outcome variable: the annual cumulative incidence of meningitis cases. Scatterplots (of each input parameter against the sensitivity outcome variable) were generated to check that the assumption of monotonicity was satisfied. The sign of the PRCC identifies the specific qualitative relation between each of the estimated parameters and the sensitivity outcome variable. We used the PRCC to identify key parameters that contributed the most to the models’ predictions imprecision.

## Results

### Model fit

The three models reproduced the weekly incidence of meningitis cases across the 64 health centre years with a good accuracy. Median *R*^2^ over all health centre years was 0.72, 0.86 and 0.87 for model1-‘inv’, model2-‘transm’ and model3-‘inv-transm’, respectively (Table 2). On average, model1-‘inv’ underestimated observed values, namely the peak incidence values (highest weekly incidence in the year) by 2%, while model2-‘transm’ and model3-‘inv-transm’ overestimated observed incidences by 5% and 1%, respectively. The error rates of the three models were relatively low but model 1-‘inv’ had an error rate (RSR = 0.52) that is about 40% higher than for model2-‘transm’ and model3-‘inv-transm’ (Table 2). Adding annual seasonality of the transmission parameter to seasonality of the invasion rate (model3-‘inv-transm’) improved the weekly incidence predictions of model1-‘inv’ overall (*R*^2^ and error rate RSR improved). However, the gain in prediction accuracy was marginal when comparing model3-‘inv-transm’ to model2-‘transm’ performances (Table 2).

**Table 2.** Quantitative performances (goodness of fit) of the three compartmental models in predicting annual seasonal hyperendemic incidence of 64 health centre years in four health districts of Burkina Faso during 2004–2010

Models	*R*^2^^a^		PB (%)^b^		RSR^c^	
	Median	1st, 3rd quartile^d^	Median	1st, 3rd quartile	Median	1st, 3rd quartile
Model1-‘inv’	0.72	0.62, 0.83	−2.30	−11.10, 4.20	0.52	0.41, 0.61
Model2-‘transm’	0.86	0.78, 0.92	0.50	−7.10, 1580	0.37	0.28, 0.47
Model3-‘inv-transm’	0.87	0.78, 0.92	4.96	−10.20, 11.20	0.36	0.28, 0.46

a *R*^2^: coefficient of determination. Refers to the variance in observed data explained by the model.

b PB: per cent bias (%). Average tendency of the simulated values to be larger or smaller than their observed ones.

c RSR: ratio of root-mean-square error (RMSE) to standard deviations of observations.

d 1st, 3rd quartiles refers to: first and third quartiles of the estimates distribution.

**Fig. 2.**
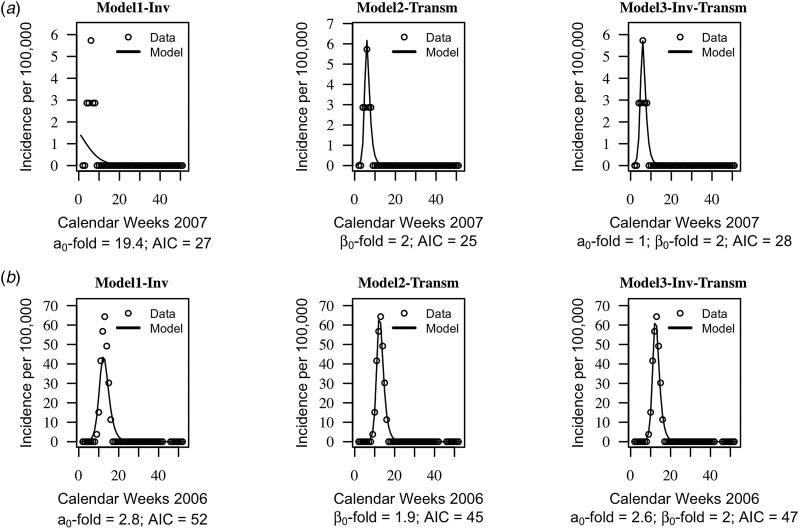
Trajectory matching plots of observed weekly incidence data and models’ predictions. Data (hallow circles) and models predictions (black solid line). (a) Health centre year with the poorest fitted data. (b) Health centre year with the best-fitted data. *a*_0_-fold and *β*_0_-fold indicate the seasonal fold increase of the invasion and transmission rate (respectively) relative to their baseline or average value. Model1-‘inv’: seasonal forcing of the invasion rate alone, model2-‘transm’: seasonal forcing of the transmission rate alone, and model3-‘inv-transm’: seasonal forcing of the transmission and invasion rate. Trajectory matching plots for all 64 health centre years are provided in Supplementary Figs S1–S3. Simulations are based on best-fit estimates of the parameters.

The AIC of the three models were on average similar, suggesting that the models cannot be distinguished based on their quantitative performance alone (mean AIC = 46, standard deviation s.d. = 19 for model1-‘inv’; mean AIC = 44, s.d. = 20 for model2-‘transm’ and mean AIC = 46, s.d. = 20). Trajectories matching plots between the models predictions of weekly incidences and data at each health centre year suggested that seasonal trends in data were captured well by the three models, but model2-‘transm’ and model3-‘inv-transm’ captured annual peaks of disease incidence better than model1-‘inv’ in some health centre years (Fig. 2, Supplementary Figs S1–S3).

Model1-‘inv’ involved an average 2.9-fold increase, s.d. = 5.5 of the baseline invasion rate, while model2-‘transm’ involved an average 2.0-fold increase, s.d. = 0.3, of the baseline transmission rate. When both seasonality of the invasion and transmission rate is included (model3-‘inv-transm’), an average 2.0-fold increase, s.d. = 1.2 of the invasion rate is involved *vs.* an average 1.6-fold increase of the transmission, s.d. = 0.3.

The weekly carriage prevalence predicted by all three models during endemic wet season were <1% and in agreement with meningococcal serogroup A carriage prevalence studies outside epidemic periods in the meningitis belt [7, 17]. During the dry season, the median value of weekly carriage prevalence peaks (across all 64 health centre years) was 12% (1st, 3rd quartile = 7%, 18%) for model1-‘inv’, 17% (1st, 3rd quartile = 13%, 26%) for model2-‘transm’ and 11% (1st, 3rd quartile = 15%, 25%) for model3-‘inv-transm’. Including age structure in the models did not improve the models fit to data nor significantly change the results. This complementary analysis and the fits results are presented in Supplementary Material S2.

### Parameter estimation

**Fig. 3.**
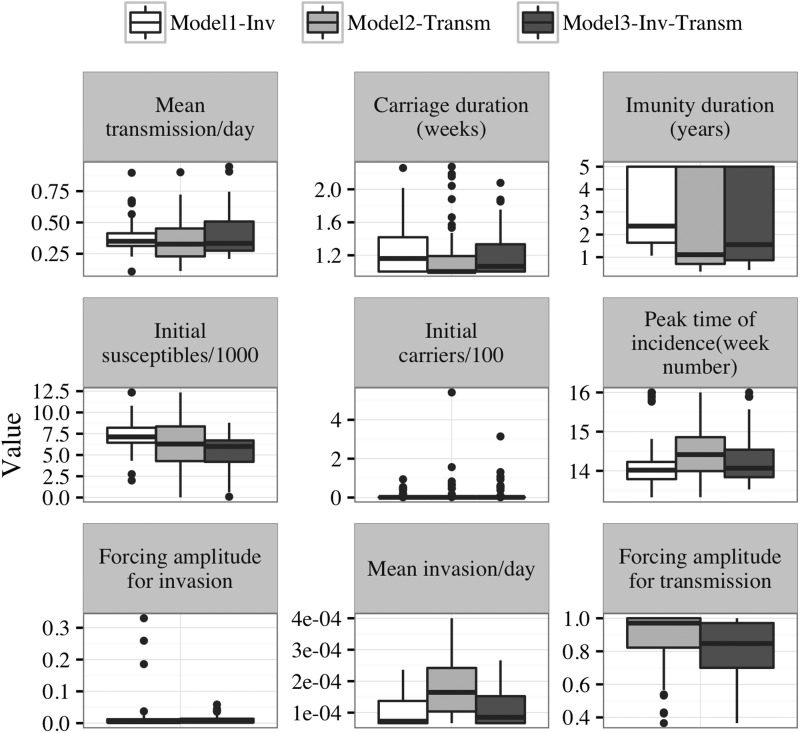
Boxplot showing the distribution of parameter estimates across all health centres years per model. The boxes include 50% of the distribution, and dots represent outliers’ values. Tick horizontal lines in the boxes represent the median value of the estimates. Values bellow the boxes are less than the 25th percentile and values above the boxes are greater than the 75th percentile of the distributions. Initial susceptibles and carriers’ populations estimates are reported as proportion of the population as of 1 January of the calendar years. Model1-‘inv’: seasonal forcing of the invasion rate alone, model2-‘transm’: seasonal forcing of the transmission rate alone, and model3-‘inv-transm’: seasonal forcing of the transmission and invasion rate.

Estimates of the baseline transmission rate were similar in the three models, as were estimates of the average duration of immunity, the timing of weekly incidence peak, and the initial susceptibles population size in model2-‘transm’ and model3-‘inv-transm’. However, with model1-‘inv’, duration of immunity tended to be longer, and the initial susceptibles population size larger (Fig. 3, Table 3). The average invasion rate estimated by model2-‘transm’ was fourfold higher than that of model1-‘inv’ and model3-‘inv-transm’. Overall, parameter estimates with model3-‘inv-transm’ had smaller between-health centres variances than with model1-‘inv’ and model2-‘transm’ (Fig. 3, Table 3). Sensitivity analyses with hyperendemic health centre years defined as 50 weekly cases per 100 000 maintained during at least two consecutive weeks did not yield substantially different results (data not shown).

**Table 3.** Quantiles of the distributions of parameters estimated across the 64 health centre years per model

Parameters	Model1-inv			Model2-trans			Model3-inv-trans		
Quantiles	25%	50%	75%	25%	50%	75%	25%	50%	75%
Baseline transmission/day (*β*_0_)	0.312	0.349	0.413	0.229	0.326	0.451	0.274	0.332	0.507
Carriage duration (weeks) (*α*)	1.002	1.1611	1.4187	1.0027	1.0027	1.190	1.0027	1.0658	1.3336
Immunity duration (years) (*φ*)	1.640	2.374	5.000	0.701	1.108	5.000	0.866	1.554	5.000
Initial susceptibles (*S*_0_)	6443.310	7128.267	8205.869	4282.658	6289.297	8356.209	4199.00	6002	6712
Initial carriers (*C*_0_)	1.000	1.000	1.251	1.000	1.000	1.558	1.000	1.000	1.201
Peak time (week number) (*θ*)	13	14	14	14	14	15	13	14	14
Seasonal forcing of invasion (*ε*_a_)	0.002	0.004	0.012	–	–	–	0.002	0.005	0.013
Baseline invasion (*a*_0_)	1 × 10^−4^	1 × 10^−4^	1 × 10^−4^	1 × 10^−4^	2 × 10^−4^	2 × 10^−4^	1 × 10^−4^	1 × 10^−4^	2 × 10^−4^
Seasonal forcing of transmission (*ε*_b_)	–	–	–	0.822	0.970	1.000	0.700	0.847	0.970

### Uncertainty and parameters sensitivity

Uncertainty analysis results (Table 4) show that the prediction precision of the three models is low due to high degree of estimation uncertainty for the baseline values of the estimated parameters. Model2-‘transm’ has the higher prediction imprecision with a larger variance of the predicted annual cumulative incidence: 6346 compared with 439 for model1-‘inv’, and 731 for model3-‘inv-transm’. Uncertainty in estimating five of the nine estimated parameters was most critical in affecting the prediction precision of the three models. The five most critical parameters were the baseline transmission and invasion rates, average duration of asymptomatic carriage, the duration of immunity to infection and disease and the initial susceptibles population size (Table 5). The effect of uncertainty of carriage duration on prediction imprecision was more important with model1-‘inv’, than with model2-‘transm’ and model3-‘inv-transm’. Parameter sensitivity ranking based on the PRCCs indicates that with model1-‘inv’, the baseline invasion rate was the most sensitive parameter, followed by the duration of asymptomatic carriage. With model2-‘transm’, the most sensitive parameters were duration of immunity to infection and disease, and the baseline invasion and transmission rate. With model3-‘inv-transm’, the baseline transmission and population immunity were the first two most critical parameters. However, initial proportion of carriers at the beginnings of the dry season also appears critical for the later (Table 5).

**Table 4.** Description of predicted annual incidence and weekly carriage prevalence (averaged over the year) using 1000 combinations of parameters values from the Latin Hypercube Sample (uncertainty analysis)

Values	Annual incidence per 100 000 inhabitants			Average weekly carriage prevalence (%)		
	Model1	Model2	Model3	Model1	Model2	Model3
Minimum	28.70	0.06	0.28	0.90	0.00	0.01
Maximum	125.4	355.0	139.0	3.8	3.7	3.5
Mean	67.0	115.0	59.0	1.9	1.6	1.8
Median	62.3	105.0	54.0	1.8	1.5	1.7
Variance	439.9	6346.0	731.0	0.3	0.7	0.6
5**th** percentile	37.70	1.50	18.00	1.10	0.02	0.70
95**th** percentile	108.8	273.0	110.0	2.7	3.1	3.2

**Table 5.** Partial rank correlation coefficients (PRCC) between the Latin Hypercube Samples of estimated parameters and the annual cumulative incidence of meningitis (sensitivity analysis)

		Model1-‘inv’		Model2-‘transm’		Model3-‘inv-transm’	
Parameter	Short description	PRCC^a^	95% Confidence interval	PRCC	95% Confidence interval	PRCC	95% Confidence interval
*β*_0_	Meningococcal mean transmission rate	0.76***	0.68–0.84	0.80***	0.75–0.86	0.91***	0.88–0.96
*a*_0_	Meningococcal mean invasion rate	0.90***	0.86–0.96	0.84***	0.76–0.94	0.81***	0.75–0.89
*α*	Rate of loss of carriage	−0.89***	−0.93 to −0.86	−0.49***	−0.65 to −0.31	−0.63***	−0.75 to −0.54
*φ*	Rate of loss of natural immunity	0.80***	0.73–0.88	0.87***	0.82–0.93	0.90***	0.87–0.95
*θ*	Calendar day of maximal invasion rate	0.18	−0.01 to 0.36	0.03	−0.17 to 0.27	−0.04	−0.26 to 0.19
*ε*_a_	Seasonal forcing amplitude of invasion rate	−0.15	−0.34 to 0.05	NA	NA	−0.025	−0.25 to 0.22
*ε*_b_	Seasonal forcing amplitude of meningococcal transmission rate	NA^b^	NA	0.18	0.03–0.37	−0.11	−0.31 to 0.11
*S*_0_	Initial susceptibles’ proportion	0.86***	0.81–0.93	0.73***	0.66–0.84	0.81***	0.74 to 0.90
*C*_0_	Initial carriers’ proportion	0.09	−0.08 to 0.33	0.11	−0.095 to 0.28	0.22*	0.04 to 0.40

a Partial rank correlation coefficients estimates are significantly different than 0 at 0.05 level (*), and <10^−10^ level (***) two-sided *P* values. They quantify the statistical relationship between each parameter and the model output.

b NA stands for not applicable to the model.

The positive value of the PRCC for the majority of the estimated parameters values implies that when the values of these input parameters increase, the future number of meningitis cases will increase. As immunity wanes quickly, the future number of meningitis cases is likely to increase. One possible way this can occur is by fast replenishment of the pool of susceptible individuals. With higher pool of susceptible individuals and lower population level immunity, comes increased likelihood of effective transmission of infection.

## Discussion

This modelling study is a first attempt to fit compartmental models based to surveillance data of suspected bacterial meningitis at a fine spatial (health centre) and temporal (weekly) scale in the African meningitis belt. Two publications, by Karachaliou *et al*. [12] (building on Irving *et al*. [10]. work), and Tartof *et al*. [11] used meningitis compartment models to evaluate long-term vaccination strategies with serogroup A conjugate vaccine. Both studies included seasonal change of the transmission and invasion rate in an age-structured model, but did not aim at comparing models with different types of seasonal forcing with regard to the transition from endemic to hyperendemic situation. Our study aimed at investigating the pathophysiology of the seasonal hyperendemicity of bacterial meningitis in this region at a fine scale, which is extraordinarily pronounced with a 10- to 100-fold increase observed every year in all districts [6, 7]. We found that compartmental models using seasonal forcing of risk of invasive disease given carriage, transmission or both, all produced seasonal disease incidence patterns consistent with the observed data, while models containing a seasonal effect on transmission improved the fit of seasonal incidence peaks. The latter finding appears to be somewhat in contrast with the hypothetical model presented by Mueller and Gessner [16]. While the three models required similar estimates of the endemic transmission rate to reproduce the observed disease incidence, the model including seasonality of transmission only (model2-‘transm’) involved a 2–4 times higher endemic invasion rate. This suggests that it is not sufficient to have higher transmission in the dry season to accurately reproduce the observed hyperendemicity, the level of meningitis disease risk given colonisation is important as well. Also, we found that seasonal change occurred in both the transmission and invasion rate in the model including seasonality of these two parameters. Our findings seem to conflict with the results from Tartof *et al*. [11] who published an age-structured model of MenA in the meningitis belt showing that observed data trends could be explained by a model with varying infection rates, but little seasonal variation in the risk of disease given colonisation. Adding a similar age-specific contact pattern to our models did not significantly change our results nor improve the fit to the data (Supplementary Material S3). The age-specific contact matrix (Supplementary Material S2) for this complementary analysis was extrapolated from Tartof *et al*.’s [11] paper and its supplemental materials, which may have its own limitations. However, discrepancies with the Tartof *et al*.’s study may be explained by differences in the spatial scale and scope of data analyses. Tartof *et al*. used data aggregated at the district or national level and aimed at explaining the occurrence of larger epidemic clusters or epidemic waves spanning several consecutive years. In contrast, our exercise aimed at studying the transition from endemic to hyperendemic situations, excluding localised epidemics detected based on high-resolution data (health centre level). The two models therefore differ in aim and spatial scale. Their use of larger scale data, i.e. district or national while we use local health centres, may prevent from accurately discriminating epidemic from regular hyperendemic events, thus mixing two distinct disease spreading mechanisms. Until appropriate contact pattern data from the meningitis belt population become available, our complementary analysis of the models including an age-structured model of transmission (Supplementary Material S2 and S3) should be considered exploratory.

The average annual carriage prevalence estimates from our models’ uncertainty analysis exceeded 1% (1.9%). Carriage prevalence studies conducted in the meningitis belt show that, outside of epidemics, MenA carriage prevalence rarely exceeds 1%. Lack of serogroup-specific surveillance data for our model estimation may explain this behaviour, and the obtained carriage estimates represent both meningococci and pneumococci, all serogroups and type combined. Carriage studies using classical swabbing and culture inoculation techniques may have also underestimated the prevalence of nasopharyngeal carriage [6, 33–35]. Seasonal variations of the transmission rate in each health centre year appear to mirror the small or absent seasonal variations of carriage prevalence observed in available epidemiological studies [17, 31].

The model including only seasonal forcing of invasion (model1-‘inv’) required a substantially longer persistence of natural immunity following carriage or disease (median = 2.5 years *vs* 1 and 1.5 years), where the few serological studies available suggest rather shorter immunity persistence [20, 24]. An additional limitation of model1-‘inv’ was its lower accuracy in reproducing annual peaks of data in several health centre years, which was improved by an additional forcing of the transmission rate. An explanation for this could be that some health centre years incidence curves were classified as hyperendemic incidence based on the epidemic threshold definition used but were small-localised outbreaks resulting essentially from an accelerated transmission of the bacteria in the community as explained in the explanatory model suggested by Mueller and Gessner. However, sensitivity analyses with a lower epidemic threshold (50 weekly cases per 100 000) did not impact the models’ results.

The fold increase of the transmission rate was not systematically higher than that of the invasion rate. It appears that both pathophysiological mechanisms are relevant and may reflect the impact that climatic conditions have on bacterial meningitis.

This study builds on the model published by Irving *et al*. [10] who investigated how well simple deterministic models were able to qualitatively reproduce the meningitis epidemiology in the African meningitis belt. Their study was limited to larger epidemic waves that are observed every 7–10 years at the national level and did not use surveillance data for parameterisation or evaluation of model performance. The authors found that the model captured the irregular pattern of meningitis epidemics qualitatively and concluded, under the assumption of an increased bacterial transmission during the dry season, that the dynamics of population immunity could explain disease dynamics. Our study focused on hyperendemic incidences during the dry season, and results from the two studies should be considered as complementary, in particular as; as suggested by Mueller and Gessner [16], hyperendemicity, localised epidemics and epidemic waves may be distinct phenomena with distinct pathophysiological and epidemiological mechanisms. However, it appears essential to use surveillance data for parameterisation and quantitative evaluation. The availability of such data at high spatial (health centre) and temporal (weekly) resolution will allow adapting our model to reproduce the occurrence of localised epidemics, epidemic waves and meningitis incidence at the regional level using meta-populations models. Eventually integrating immunisation interventions, such models will serve to develop optimised vaccination strategies against meningococcal and pneumococcal meningitis. We identified key parameters for which more data from clinical and epidemiological studies are needed to improve prediction, in particular duration of immune protection and carriage episodes, rates of invasion and transmission of the bacteria, and their variation by season.

Our study has some limitations inherent to the deliberately simple model structure and assumptions. We assumed that mixing among individuals was homogeneous. Meningococcal carriage and disease affect different age groups at different rates [31] and it is expected that contacts will be more intense between individuals in the same age group, in particular for older children and young adults. Limitations inherent to our extrapolation of age-specific contact pattern from Tartof *et al*.’s paper may have prevented our age-structured model from achieving better fit to the data than the simpler model. Similarly, we assumed only one level of protection against carriage and disease, given the sparsity of evidence, while models evaluating vaccination strategies will require more distinct assumptions.

We used sinusoidal functions to force the seasonality of the transmission and invasion parameters, while an improved approach could consist in modelling these two parameters as a function of climatic variables, such as mean aerosol load, that are known to correlate well with seasonal meningitis incidence [36–38]. In some health centres with small population size, we had to limit the effect of random noise in the data by smoothing the time series to focus on the underlying seasonal trend. Chance variations of some unknown parameters, in particular the extent of climate conditions changing from year to year, was not explicitly included in the model structure. We addressed this in part by fitting the parameters on a yearly basis rather than using a single multiple year time series. However, stochastic models may be more appropriate when these fluctuations are important. Stochastic models shall be explored in the future for they appear to be particularly relevant when modelling localised epidemics. We used a model structure of overall meningococcal carriage and infection. The epidemiology of carriage likely differs between meningococcal and pneumococci meningitis but the limited knowledge about both bacteria dynamics made it challenging to adapt the proposed model to include pneumococci carriage data. Finally, our analysis carried on hyperendemic bacterial meningitis, i.e. both meningococcal and pneumococcal meningitis, assuming similar pathophysiologic mechanisms [39]. This assumption may not hold with regard to a variety of factors, including age structure of carriage, duration of carriage and immunity. However, given the lack of pathogen-specific meningitis surveillance data over a long period and in a large area, our approach appears justified, while it should be improved as appropriate surveillance data become available.

Despite these limitations, our findings suggest that the ubiquitous hyperendemicity of bacterial meningitis during the dry season in the African meningitis belt occurs due to a combination of increased risk of meningitis given asymptomatic carriage and meningococcal transmission. Despite the description of this phenomenon by Lapeyssonie [40] more than 50 years ago, the biological mechanisms for this pronounced seasonality remain largely unknown and little is known about the impact of aerosols and low air humidity on the human mucosal structures, immune system and interaction with the bacteria.

## Supplementary material

For supplementary material accompanying this paper visit https://doi.org/10.1017/S0950268818002625. click here to view supplementary material
