# Supplementary material for: Compartmental models for seasonal hyperendemic bacterial meningitis in the African meningitis belt
Source: Epidemiol Infect. 2018 Sep 28;147:e14. doi: 10.1017/S0950268818002625 (PMC6520558; doi:10.1017/S0950268818002625)
Supplement: Supplementary file 1 [file S0950268818002625sup001.zip › S0950268818002625sup001/koutangni_supplementary_material_S1.docx]

## Epidemiology and Infection

## Compartmental Models for Seasonal Hyperendemic Bacterial Meningitis in the African Meningitis Belt

## Thibaut KOUTANGNI, Pascal Crépey, Maxime Woringer, Souleymane Porgho, Brice Wilfried Bicaba, Haoua Tall, Judith E. Mueller

## Supplementary Material S1. Model Fitting and Parameters Estimation.

We used maximum likelihood approach to numerically estimate the models unknown parameters and reproduce the observed trend in bacterial meningitis.

For each model, we compute the log likelihood of the data given the model predictions and its parameters. Parameters were choosing to maximize the Poisson Log-likelihood (logL) of observed data series.

Given a set of N data points representing weekly number of reported meningitis cases, k_w_ (with w = 1, 2 … N) by a given health center and year, the probability or likelihood *L* of observing those data points with model predictions for each point, λ_w_, is:

|  | $L=\prod_{w=1}^{N} \frac{\lambda_{w}^{k_{w}}e^{{-\lambda}_{w}}}{k_{w}!}$ | (1) |
| --- | --- | --- |

The log-likelihood to maximize was therefore defined as

|  | $logL=\sum_{w=1}^{N} \left( k_{w} log\lambda_{w}-\lambda_{w} \right)$ | (2) |
| --- | --- | --- |

Again, k_w_ and λ_w_ are the observed and simulated cases for week w respectively, and N is the number of weeks of the calendar year (typically 52 or 53).

The process of finding the set of parameters values that maximize the Poisson Log-likelihood of observed data was conducted using the COBYLA algorithm, a derivative-free optimization algorithm (implemented in R package nloptr [1, 2]) which allows setting lower, and upper bounds on the parameters space to search as well as nonlinear constraints. We defined a constraint on the average magnitude of change of carriage prevalence between the wet endemic and dry hyperendemic season, to reflect that observed in a carriage study [3]. We also set lower and upper bounds on the parameter space to search based on the scientific literature if possible. Because the COBYLA algorithm implementation was design to minimize an objective function, we rather minimized the negative log likelihood (-logL), which is equivalent to maximizing the logL.

|  | $-logL=\sum_{w=1}^{N} \left( -k_{w} log\lambda_{w}+\lambda_{w} \right)$ | (3) |
| --- | --- | --- |

We run the 3 models separately with each of the health center-year data. An optimal solution was reached before the set maximum number of iterations (40000). We then simulate each model with its best-fit parameters estimates and compare it predictions of weekly cases with the health center-year weekly incident cases reports.

## Models performances and comparison.

To evaluate how well each model performs in predicting a given health center year incidence data we used the following criteria.

The coefficient of determination (R^2^ ). This quantity measured the amount of variance in the health center-years data explained by a given model.

|  | $R^{2}=1-\frac{\sum_{i=1}^{n} \left( y_{i}-\hat{y}_{i} \right)^{2}}{\sum_{i=1}^{n} \left( y_{i}-\bar{y}_{i} \right)^{2}}$ | (4) |
| --- | --- | --- |

$y_{i}$ is the observation data point, $\hat{y}_{i}$ its predicted value, and $\bar{y}_{i}$ the mean of the n observation data points.

The percent bias (PBIAS) measures the average trend of simulated values to be smaller or larger than their observed ones. The optimal value of PBIAS is 0.0, with low-magnitude values indicating accurate model simulation. Positive values indicate overestimation bias, whereas negative values indicate model underestimation bias [4].

|  | $PBIAS=\left( \frac{\sum_{i=1}^{n} \left( \hat{y}_{i}-y_{i} \right)(100)}{\sum_{i=1}^{n} \left( y_{i} \right)} \right)$ | (5) |
| --- | --- | --- |

Another model evaluation statistic used was the Ratio of the Root Mean Squared Error between simulated and observed values to the standard deviation of the observations (RSR). RSR standardizes the Root Mean Squared Error using the observations standard deviation, and has the benefits of combining both an error index and scaling/normalisation factor (Legates and McCabe, 1999). RSR varies from the optimal value of 0.0, which indicates zero RMSE and therefore perfect model simulation, to a large positive value. The lower RSR, the lower the RMSE, and the better the model simulation performance.

|  | $RSR=\frac{\left[ \sqrt{\sum_{i=1}^{n} \left( y_{i}-\hat{y}_{i} \right)^{2}} \right]}{\left[ \sqrt{\sum_{i=1}^{n} \left( y_{i}-\bar{y}_{i} \right)^{2}} \right]}$ | (6) |
| --- | --- | --- |

To compare and determine which model was most realistic regarding it’s ability to reproduce observed meningitis cases reports while accounting for model complexity, we computed the Akaike Information Criteria (AIC). The AIC was computed as follows: AIC = 2p - 2ln(L) where p is the number of estimated parameters of the model and L the maximum likelihood. The lower the AIC the better the model. As a rule for decision we considered a is model significantly different than another if the absolute difference in their AIC is at minimum of 2 units.

References

1. Powell MJD. A direct search optimization method that models the objective and constraint functions by linear interpolation. Adv. Optim. Numer. Anal. 1994;51–67.

2. Johnson SG. The NLopt nonlinear-optimization package. Available from: http://ab-initio.mit.edu/nlopt

3. Kristiansen P a, Diomandé F, Wei SC, et al. Baseline meningococcal carriage in Burkina Faso before the introduction of a meningococcal serogroup A conjugate vaccine. Clin. Vaccine Immunol.. 2011;18(3):435–43.

4. Mauricio Z-B. Goodness-of-fit functions for comparison of simulated and observed hydrological time series. 2014. Available from: http://cran.r-project.org/package=hydroGOF
