# Supplementary material for: Compartmental models for seasonal hyperendemic bacterial meningitis in the African meningitis belt
Source: Epidemiol Infect. 2018 Sep 28;147:e14. doi: 10.1017/S0950268818002625 (PMC6520558; doi:10.1017/S0950268818002625)
Supplement: Supplementary file 1 [file S0950268818002625sup001.zip › S0950268818002625sup001/koutangni_supplementary_material_S2.docx]

## Epidemiology and Infection

## Compartmental Models for Seasonal Hyperendemic Bacterial Meningitis in the African Meningitis Belt

## Thibaut KOUTANGNI, Pascal Crépey, Maxime Woringer, Souleymane Porgho, Brice Wilfried Bicaba, Haoua Tall, Judith E. Mueller

**Supplementary Material** **S2.** **Supporting information:** **Age-specific contact matrix.**

We included age structure in the SCIRS model and simulated the three models in a complementary analysis of the behavior of our models when including heterogenous mixing. The age-structured model, considered four age groups based on available age-specific force of infection estimates from a published modeling study using data from the meningitis belt[1]. These age groups were: <5 years, 5-12 years,13-19 years, 20 years and older. The contact matrix C between the defined 4 age groups infered based on the published age-specific estimates of the force of infection during dry season

(minor epidemic) was as follows:

|  | | Age of infectious (j) | | | |  |
| --- | --- | --- | --- | --- | --- | --- |
|  |  | <5 | 5-12 | 13-19 | 20+ |  |
| Age of susceptible (i) | <5 | 0.176 | 0.074 | 0.050 | 0.061 |  |
|  | 5-12 | 0.200 | 0.064 | 0.071 | 0.059 |  |
|  | 13-19 | 0.116 | 0.358 | 0.250 | 0.108 |  |
|  | 20+ | 0.068 | 0.137 | 0.080 | 0.336 |  |
|  |  |  |  |  |  |  |
|  |  |  |  |  |  |  |

The calculated numbers represent rates at which individuals in the ith age group comes onto effective contact with individuals in the jth age-group per day. Studies reporting typical contact patterns in the meningitis belt are lacking at the time of writing, and we attempted, in this complementary analysis, to extrapolate a possible contact matrix from [1]. Until appropriate data becomes available in the meningitis belt the results from using this contact matrix should be considered explorarory. Trajectories matching plots of the age structured models predictions and data are as well as statistics of the goodness of fit are provided in Supplementary Material S3.

References:

1. Tartof S, Cohn A, Tarbangdo F, Djingarey MH, Messonnier N, Clark TA, et al. Identifying Optimal Vaccination Strategies for Serogroup A Neisseria meningitidis Conjugate Vaccine in the African Meningitis Belt. PLoS One. 2013;8(5):e63605.
