# Supplementary figures and images for: Compartmental models for seasonal hyperendemic bacterial meningitis in the African meningitis belt
Source: Epidemiol Infect. 2018 Sep 28;147:e14. doi: 10.1017/S0950268818002625 (PMC6520558; doi:10.1017/S0950268818002625)

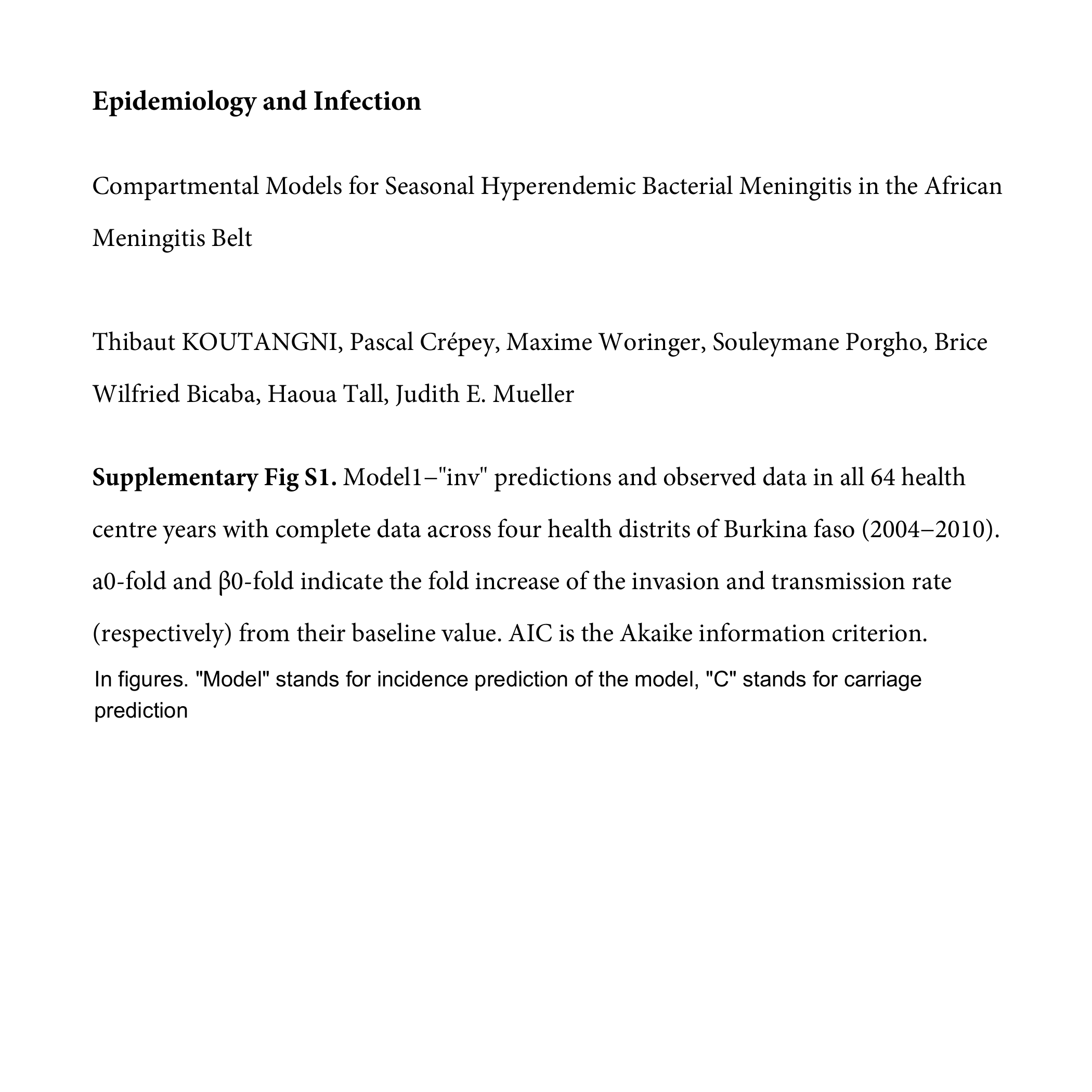

Supplement: Supplementary file 1 [file S0950268818002625sup001.zip › S0950268818002625sup001/koutangni_et_al_supplementary_fig_S1.tif]

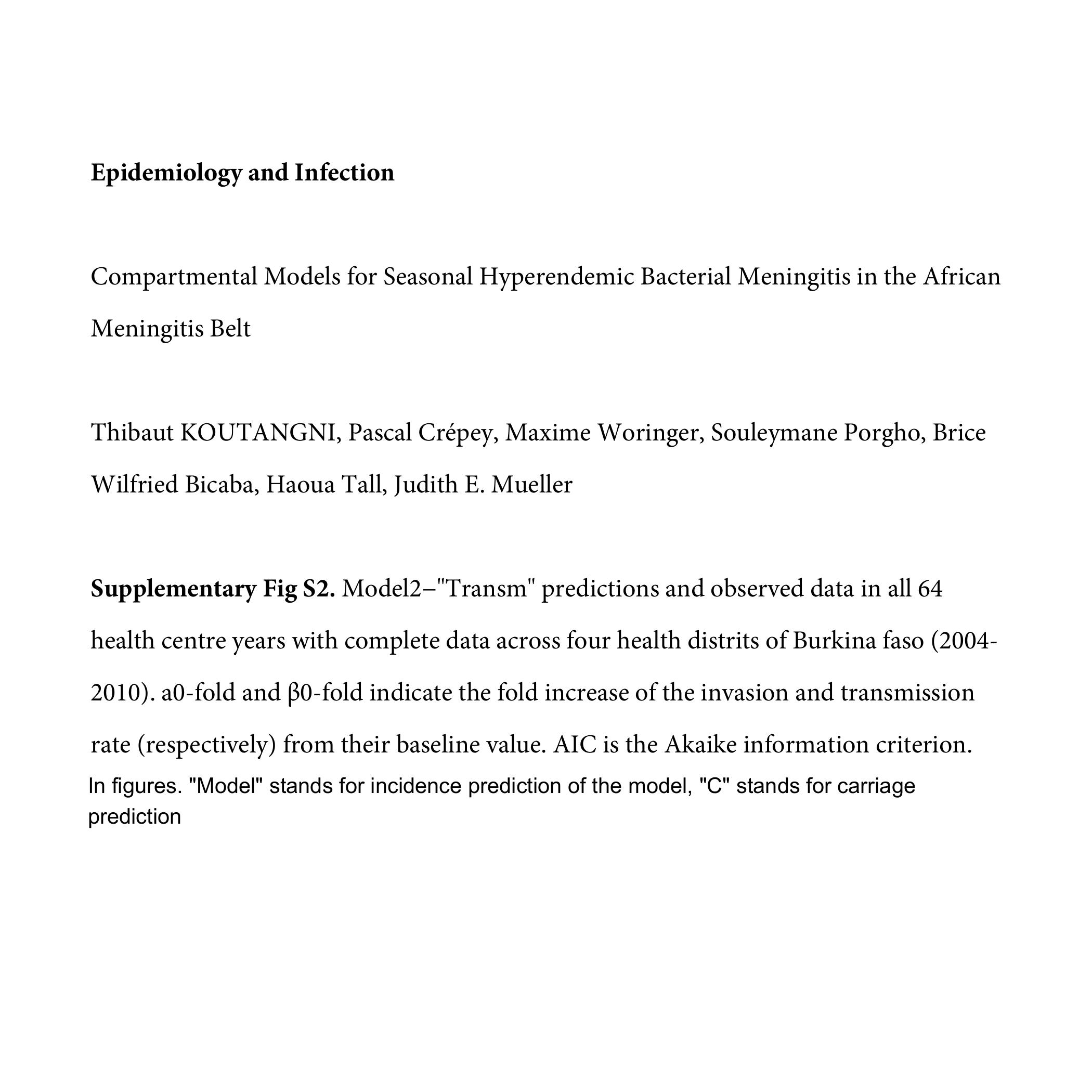

Supplement: Supplementary file 1 [file S0950268818002625sup001.zip › S0950268818002625sup001/koutangni_et_al_supplementary_fig_S2.tif]

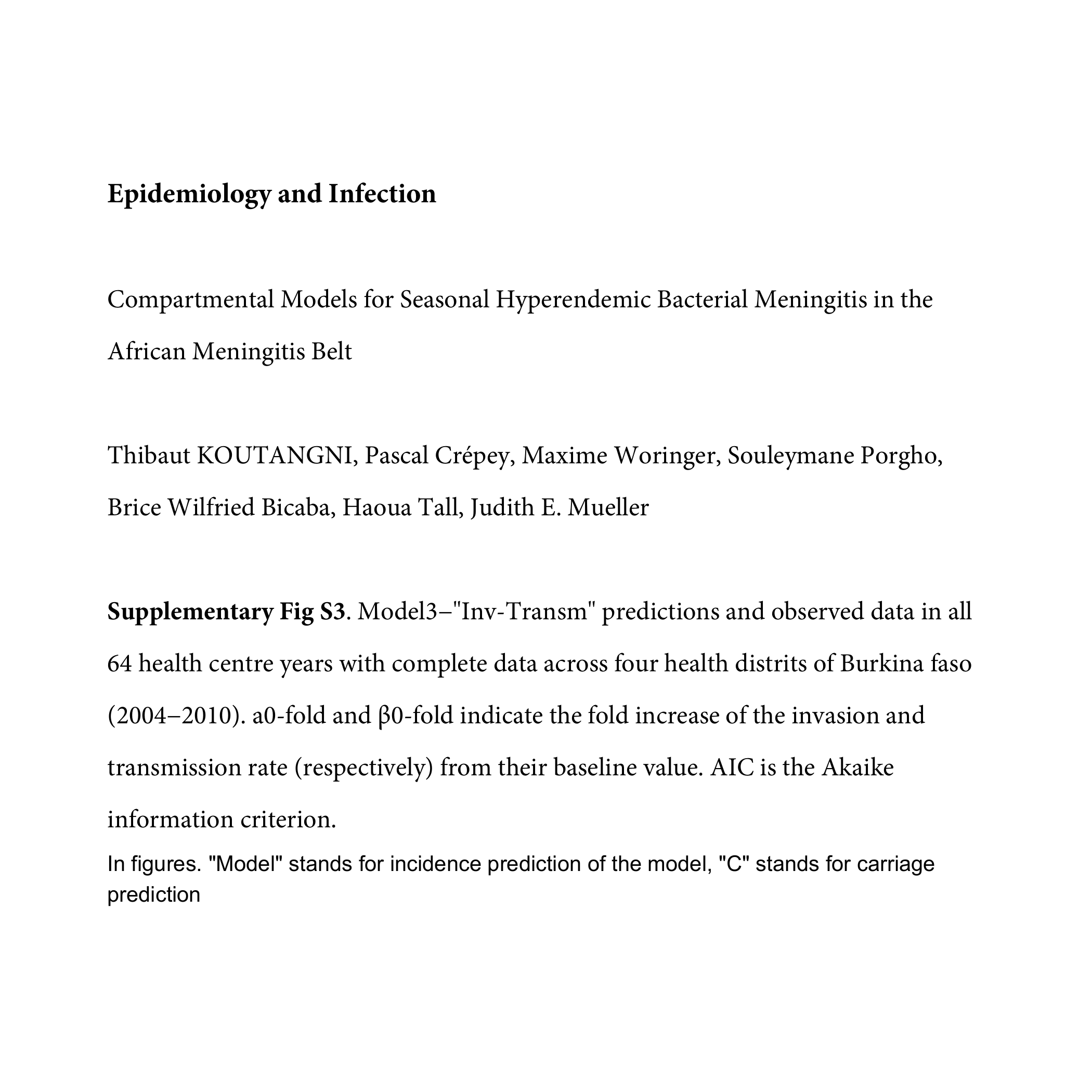

Supplement: Supplementary file 1 [file S0950268818002625sup001.zip › S0950268818002625sup001/koutangni_et_al_supplementary_fig_S3.tif]

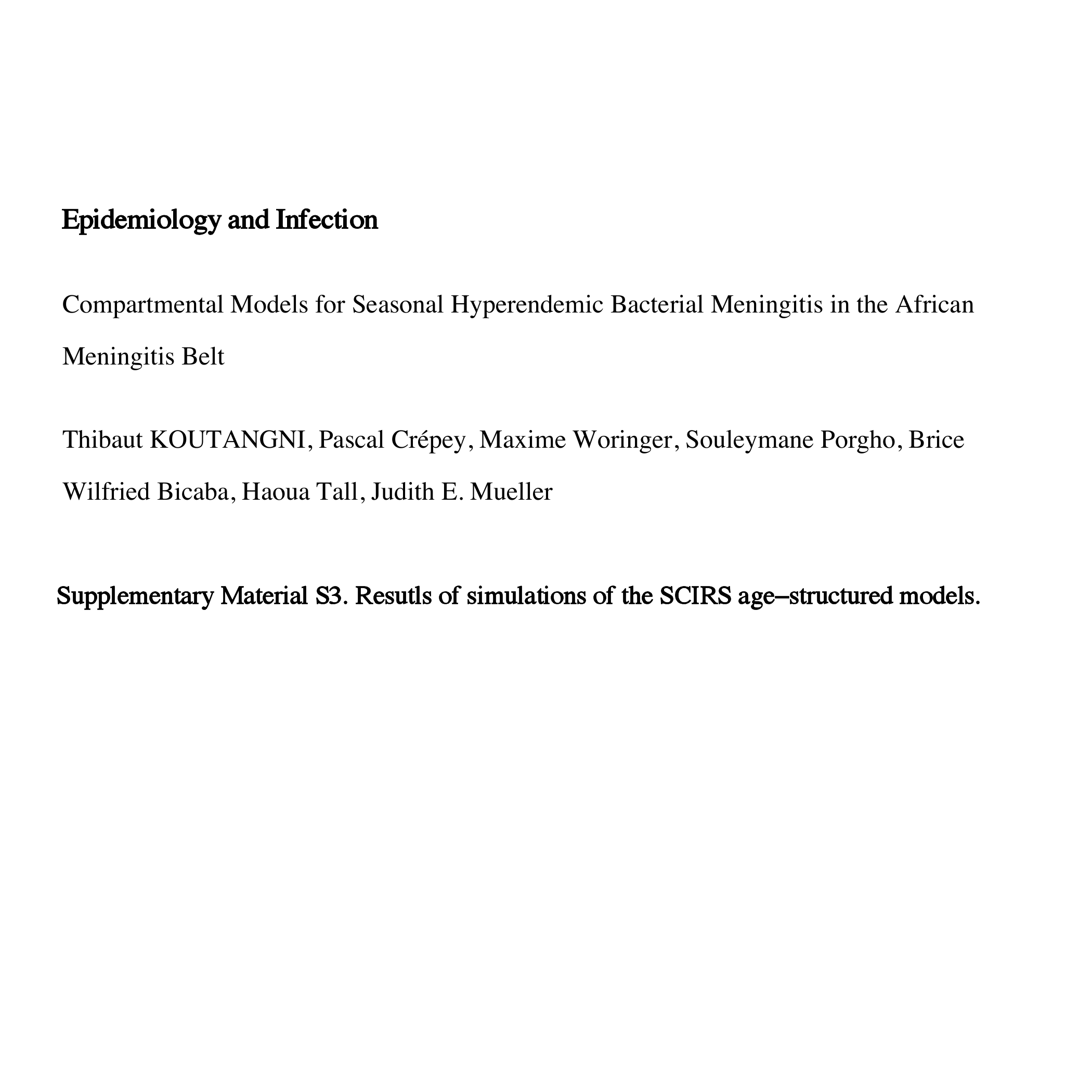

Supplement: Supplementary file 1 [file S0950268818002625sup001.zip › S0950268818002625sup001/supplementary_material_S3.tif]
